# An Adaptive Superpixel Based Hand Gesture Tracking and Recognition System

**DOI:** 10.1155/2014/849069

**Published:** 2014-05-27

**Authors:** Hong-Min Zhu, Chi-Man Pun

**Affiliations:** Department of Computer and Information Science, University of Macau, Macau

## Abstract

We propose an adaptive and robust superpixel based hand gesture tracking system, in which hand gestures drawn in free air are recognized from their motion trajectories. First we employed the motion detection of superpixels and unsupervised image segmentation to detect the moving target hand using the first few frames of the input video sequence. Then the hand appearance model is constructed from its surrounding superpixels. By incorporating the failure recovery and template matching in the tracking process, the target hand is tracked by an adaptive superpixel based tracking algorithm, where the problem of hand deformation, view-dependent appearance invariance, fast motion, and background confusion can be well handled to extract the correct hand motion trajectory. Finally, the hand gesture is recognized by the extracted motion trajectory with a trained SVM classifier. Experimental results show that our proposed system can achieve better performance compared to the existing state-of-the-art methods with the recognition accuracy 99.17% for easy set and 98.57 for hard set.

## 1. Introduction

Being a significant part in interaction of communication in our daily life (human-human or human-computer), hand gestures provide us a natural and user friendly way of interaction. With the progress of gesture tracking and recognition techniques, the computer vision field has experienced a new opportunity of applying a practical solution for building a variety of systems [1, 2] such as surveillance, smart home, and sign language recognition. Early systems that make use of gestures as interaction usually require an additional pointing device (e.g., data gloves and markers) to detect the movement; these sensor-based solutions can provide accurate measurements of hand pose and movement while they require extensive calibration, restrict natural hand motion, and are usually expensive. Recent systems focused on gestures performed by hand freely in 3D space without any physical attachments, and gestures are captured by various cameras which are analyzed and recognized with video-based solutions. Locating the hands and segmenting them from the background usually encounter difficulties when there are occlusions, lighting variances, fast motion, or other objects present with similar appearance. There are many vision-based hand gesture recognition algorithms proposed in past several decades which attempted to provide robust and reliable systems, as reviewed in [3, 4]. The common methods for hand detection are skin-color maps [5] and cascaded classifiers on Haar-like features [6]. Skin-color based approaches may be easily affected by lighting changes. Another set of hand detection approaches are clustering [7] and region growing [8] which are both time consuming processes. The hand tracking solution can benefit from visual object tracking solutions [9–13] which are based on cues ranging from low-level visual features to high-level structural information. The PROST method [9] extends the idea of tracking-by-detection such as [10] with multiple modules to reduce the drifts and object deformation; however the tracker is easily distracted by object with similar appearance. The visual tracking decomposition approach (VTD) [11] gets the tracking result with significant amount of noise from the background patches which combined particle filter with multiple observation and motion models; the tracker encounters failures when distinguishing the target object and its background. Spatiotemporal structural context based tracker (STT) [12] captured the historical appearance information to prevent the target object from drifting to the background in a long sequence; the supporting field built from spatial contributors provides more information to predict the target. Another potential solution is superpixel tracking (SPT) [13], which used mid-level clustering of histogram information captured in superpixels and a discriminative appearance model formulated with target-background confidence map, which tried to find proper appearance models that distinguish one object with all other targets or background. However, this approach is not very reliable when severe deformation or background confusion exists. In the area of hand gesture recognition, there are less works relayed on hand's motion trajectories, compared to gestures represented by palm and finger's appearance and motions. Alon et al.'s work [1] proposed a classifier-based pruning framework for early rejecting of the poor matches, and a subgesture reasoning algorithm to identify falsely matched parts in longer gestures; however they detect the hand location in each frame independently with color and motion information and the appearance changes are not adaptively learnt, the multiple hand region candidates may cause confusion between the palm and the arm.

**Figure 1:**
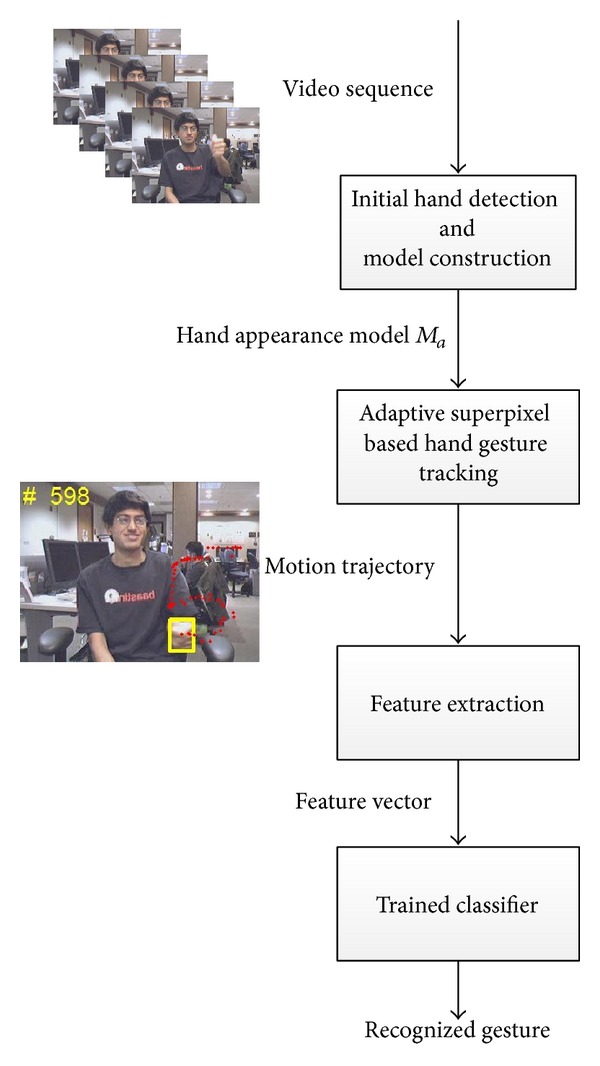
Overall framework of the proposed hand gesture recognition system.

In this paper an adaptive superpixel based hand gesture tracking and recognition system was proposed, in which hand gestures drawn in free air are recognized from the extracted motion trajectory. The overall system framework is shown in Figure 1. With the given input video sequence, the moving target hand is first detected to construct its appearance model by the proposed Initial Hand Detection and Model Construction algorithm using the first few video frames. Then the hand gesture motion trajectory is tracked by the proposed Adaptive Hand Gesture Tracking algorithm. Finally the normalized B-Spline feature vector is extracted from motion trajectory and fed to a trained SVM classifier to output recognized hand gesture. The rest of the paper is organized as follows. In Section 2 we describe the details of our proposed Initial Hand Detection and Model Construction algorithm. In Section 3, the proposed Adaptive Hand Gesture Tracking algorithm will be described. Then the procedure of feature extraction and classification is introduced in Section 4. Experimental results are given and discussed in Section 5, and finally the conclusions are drawn in Section 6.

## 2. Initial Hand Detection and Model Construction

**Figure 2:**
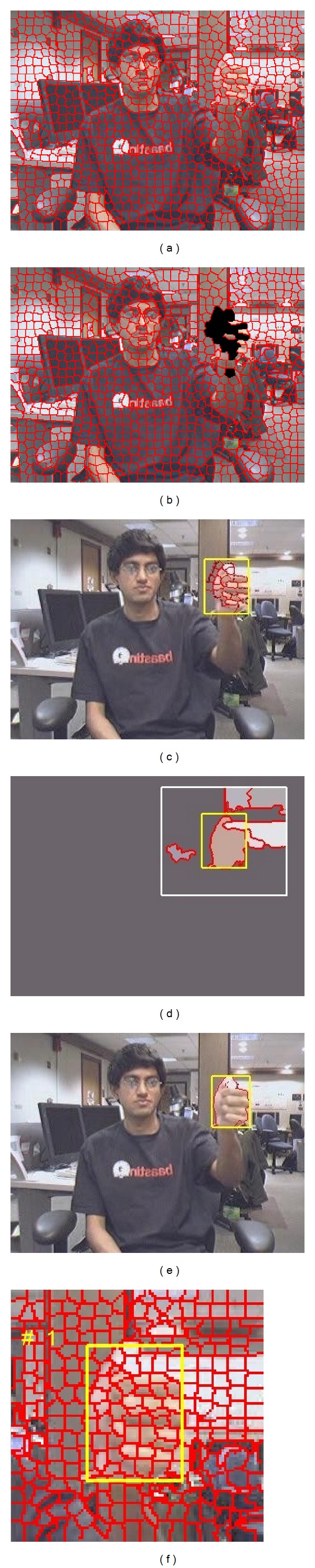
SLIC and CTM hand detection. (a) SLIC superpixels on the first frame. (b) Superpixels with slight motions. (c) Candidate hand region on the connected superpixels. (d) CTM objects on the surrounding of candidate hand region. (e) Refined hand region. (f) Superpixel on the surrounding of the hand region.

As shown in Figure 1, the first step of our proposed hand gesture recognition system is to detect the moving target hand and construct its appearance model. In order to locate the position of the moving target hand, we employed the motion detection of superpixels and unsupervised image segmentation on the first few frames of the input video sequence. The simple linear iterative clustering (SLIC) superpixels [14] solution has been widely used in the area of image segmentation and object recognition with some good results; the method over-segments the image into numerous superpixels of which object regions are composed, and the boundaries are not significantly destroyed. We employed the SLIC superpixel as the slight hand motion, which can be detected from corresponding superpixels changes in between adjacent frames. The first frame *I*_1_ is segmented into *P* superpixels *S*_*p*_ (Figure 2(a)), from which the object boundaries are approximated. The accumulated intensity changes *D*_*p*_ of each superpixel *S*_*p*_ between *I*_1_ and *I*_*i*_ can be computed as

(1) $$\begin{array}{c} D_{p}=\overset{M}{\underset{i=2}{\sum }}\left|I_{i}\left(S_{p}\right)-I_{1}\left(S_{p}\right)\right|,\;p=1,\ldots ,P. \end{array}$$

And the slight motion of a superpixel is detected (Figure 2(b)) if

(2) $$\begin{array}{c} \frac{D_{p}}{\left|S_{p}\right|}>T_{0}, \end{array}$$

where *T*_0_ is a threshold of the normalized distance and |*S*_*p*_| is the size of *p*th superpixel. After we merged neighbored superpixels with intensity changes as *R* candidate regions of the hand (Figure 2(c)), we used the compression-based texture merging (CTM) [15] based image segmentation to select the hand region from candidates. CTM used lossy compression-based clustering of texture features for the superpixels which are merged to form the object regions. The texture is modeled with a mixture of Gaussian distributions which can be degenerated; the approach shows precise segmentation on various images. We used the SLIC superpixel approach instead of the superpixel solution used in CTM. We get the *K* CTM object regions *O*_*k*_ with areas *A*_*k*_  (*k* = 1,…, *K*) on the surrounding of candidate hand region (twice the area size) in the first frame (Figure 2(d)), and the region with maximum percentage of the region area overlapped with hand candidates *R* is stated as detected hand *R*_*H*_ (Figure 2(e)):

(3) $$\begin{array}{c} R_{H}=O_{k}\;|\;A_{k}=\max\left(\frac{\text{size}\left(R\cap O_{i}\right)}{\text{size}\left(R\cup O_{i}\right)}\right),\;i=1,\ldots ,K. \end{array}$$

As we can see from the example, motion detection based on SLIC superpixels locate the hand region as shown in Figure 2(c), which include the region besides the left side of the hand since the hand moves from right to left in this case. The result is then refined by CTM segmentation to exclude the false region part. The initial hand detection is represented by a bounding box of the hand region in the first frame, although the motion information with changed intensity is accumulated from the first *M* frames.

With the gesture hand detected in the first frame, we use a simple strategy to track the hand in first *M* frames (except the first frame) and construct an initial hand appearance model. Let *X*_*t*=1_ be the hand location in the first frame (Figure 2(e)) which is represented by center of the hand region and its scale; we sample *N* hand candidates around *X*_*t*=1_ in each frame *t*  (*t* = 2,…, *M*) and the similarity between each candidate *X*_*t*_^*n*^  (*n* = 1,…, *N*) and *X*_*t*=1_ is

(4) S(X1,Xtn)=S(X1,Xtn)∑i=1NS(X1,Xti),where S(X1,Xtn)=exp⁡(−∑(I1−Itn)2c),

where *I*_*t*_^*n*^ is the grayscale image patch of *X*_*t*_^*n*^, and *c* is the condensation constant parameter. The hand detection *X*_*t*_ is selected with maximal similarity. Then SLIC segmentation on the surrounding region of *X*_*t*_ gets the *P*_*t*_ superpixels (as in Figure 2(f)) in and the *YCbCr* histogram *f*_*t*_ of each superpixel is calculated; here surrounding region is a square area centered at the same location as *X*_*t*_ and with size greater than *X*_*t*_. Our targeted hand gestures are captured in in-door environment that the color appearance of the hand is greatly affected by lighting changes which makes the feature of the hand unstable. The *YCbCr* color space encodes the illumination information in the separated component *Y*, which reduces the lighting problem by using the only *Cb* and *Cr* components. The accumulated feature set {*f*_*t*_^*r*^}_*r*=1_^*P*^ from *M* frames is clustered with mean shift clustering. The initial appearance model is then trained by calculating the target-background confidence for each cluster *i*:

(5) $$\begin{array}{c} C_{i}^{c}=\frac{\text{Siz}{\text{e}}^{+}\left(i\right)-\text{Siz}{\text{e}}^{-}\left(i\right)}{\text{Siz}{\text{e}}^{+}\left(i\right)+\text{Siz}{\text{e}}^{-}\left(i\right)},\;\forall i=1,\ldots ,n, \end{array}$$

where Size^+^ is the size of cluster *i* overlapping the object (area of *X*_*t*_) and Size^−^ is the size of *i* outside the object. Finally the hand appearance model is measured by cluster confidence *C*_*i*_^*c*^, cluster centers *f*_*c*_(*i*), cluster radius *r*_*c*_(*i*), and cluster members {*f*_*t*_^*r*^ | *f*_*t*_^*r*^ ∈ *i*}.

The initial hand detection and model construction procedure is summarized in Algorithm 1.

## 3. Adaptive Superpixel Hand Gesture Tracking

**Figure 3:**
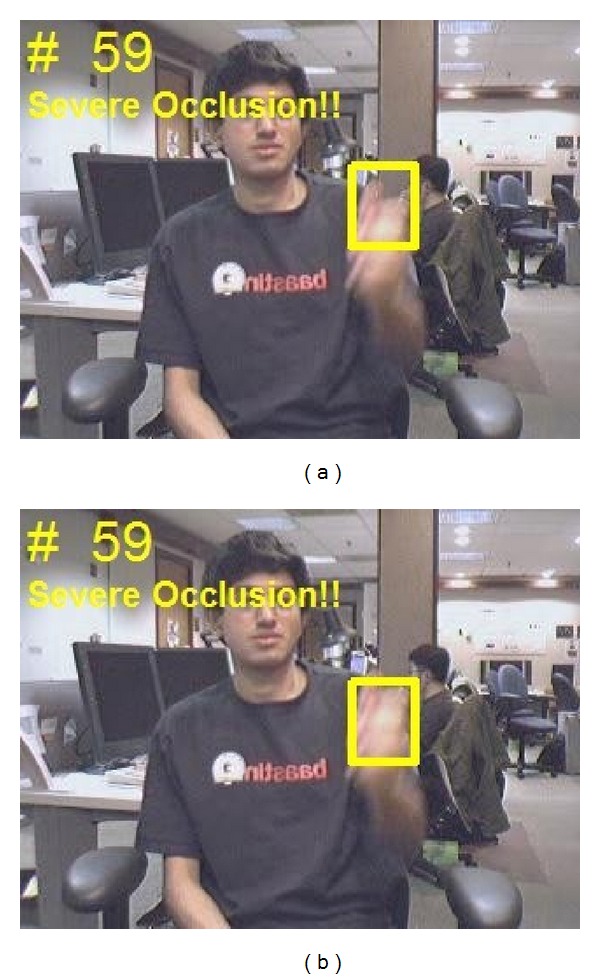
Typical example results. (a) Occlusion occurred in SPT; (b) occlusion recovered in our hand tracking solution.

**Figure 4:**
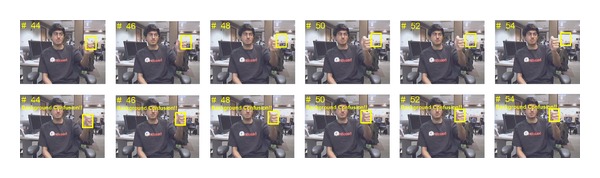
Typical example results. First row: background confusion occurred in SPT from frame 44 to frame 54. Second row: background confusion recovered in our hand tracking solution.

**Figure 5:**
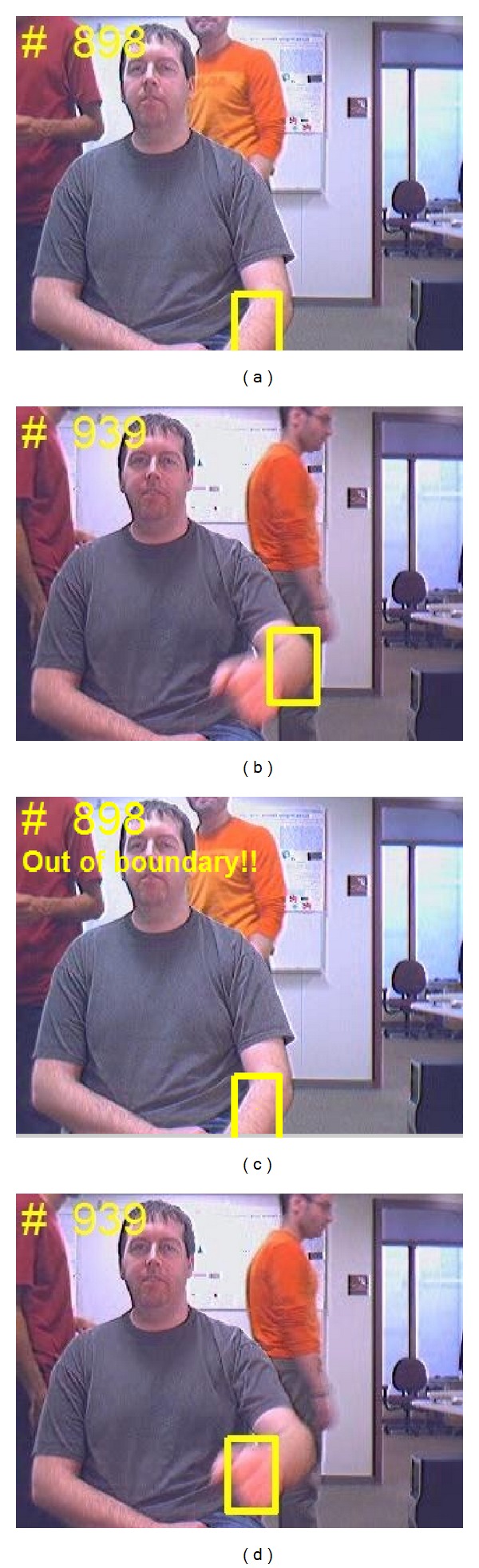
Typical example results. (a) Hand region disappeared in the scene. (b) Hand region tracked after it reappeared in SPT. (c) Detect the disappearance of hand region in our solution. (d) Hand region tracked after it reappeared in our solution.

After the initial hand appearance model is constructed from the first few frames, the positions of the target hand need to be tracked in following video frames to obtain the motion trajectory for gesture classification. Object tracking has been widely studied [9–13] in the past decade with successful results. However, these tracking techniques are not very robust for hand tracking, especially when there exist hand deformation, appearance changes, fast motion, and background confusion. In order to tackle these problems, we employed an adaptive superpixel based hand gesture tracking approach. The existing superpixel tracking (SPT) method [13] proposed for general object tracking frequently encounters failures in our hand gesture tracking task. Figures 3, 4, and 5 give some typical examples that SPT fails to track the gesturing hand. We state that the* occlusion* in Figure 3(a) occurred when the match scores between the candidate hand region and the hand model below a threshold, which may be caused by hand deformation and blur of fast motion, but not necessarily by overlapping with other objects. The model updating strategy of SPT considers the contents inside the tracked hand region as foreground, which may introduce false information to the updated model when occlusion occurred. The first row in Figure 4 gives an example that SPT detects the background as the hand region when it is skin-color like. If the problem continuously appears, the appearance model will eventually be updated with features extracted from the background. The model cannot be recovered as the subsequent tracking will surely label the background as the target. We consider this problem as* background confusion*. Figures 5(a) and 5(b) show the example that if the target hand disappeared in the scene for a long period, the model will be updated with false information which is similar to* background confusion*, and the subsequent hand tracking will fail. Our proposed adaptive hand tracking solution recovers from these failures to provide reliable tracking results. Hand region candidates are prerefined by incorporating domain specific knowledge so that the retracking with template matching detects the hand more accurately.

**Figure 6:**
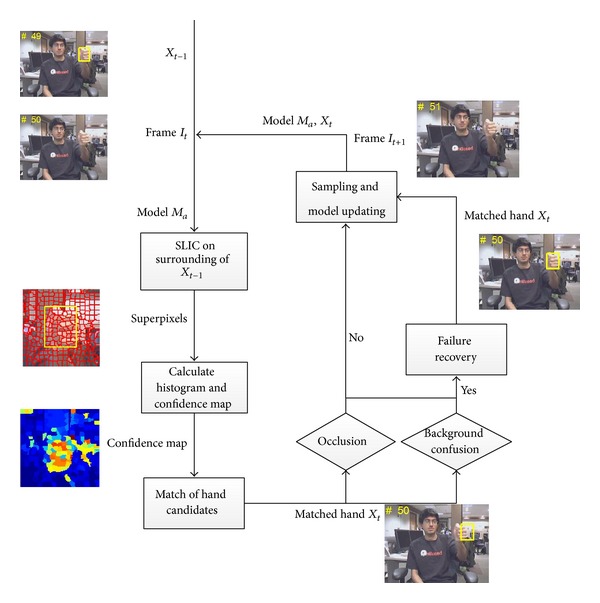
Workflow of the proposed adaptive superpixel based hand tracking.

In order to tackle the difficulties of hand deformation caused by the fast hand motion and confusion caused by background, we propose an adaptive superpixel based hand gesture tracking algorithm.  Figure 6 summarizes the workflow of our proposed algorithm. Firstly we select hand detection from candidates by matching to the initial/updated model, in case any failure occurred as introduced in Figures 3, 4, or 5, we recover and retrack the hand with template matching to give positive detections. The detected hand will be continuously and periodically sampled and used to update the hand appearance model.

From frame *t* = *M* + 1, the surrounding region of *X*_*t*−1_ is firstly segmented into *P* superpixels *S*_*p*_, and then the confidence map *C*_*r*_^*s*^ of each superpixel *r* can be computed from its histogram *f*_*t*_^*r*^ of *YCbCr* and clusters in the model:

(6) ω(r,i)=exp⁡(−2×||ftr−fc(i)||2rc(i)),∀r=1,…,Pt; i=1,…,n,Crs=ω(r,i)×Cic, ∀r=1,…,Pt,

where *f*_*c*_(*i*) is the feature center of the cluster *i* that superpixel *r* belongs to, and *r*_*c*_(*i*) is the radius of feature space of cluster *i*.

We sample *N* hand candidates around *X*_*t*−1_ and we discard those candidates that the contents of samples are occupied by non-skin-like objects:

(7) $$\begin{array}{c} \frac{\sum SK_{t}}{\text{size}\left(SK_{t}\right)}<a,\;SK_{t}=\left(B_{t}^{c}\in R_{s}\right), \end{array}$$

where *R*_*s*_ is the interval of skin color region that is defined by a Gaussian model in *YCbCr*, *SK*_*t*_ is the binary skin image of *c*th sample candidate bounding box *B*_*t*_^*c*^, and *a* is a threshold. We also discard candidates that there's no object motion detected inside the regions compared to previous frame:

(8) $$\begin{array}{c} \frac{\sum S_{t}}{\text{size}\left(S_{t}\right)}<b,\;S_{t}=x\;\;\text{or}\left(S_{t},S_{t-m}\right)\&\left(\sim S_{t-m}\right), \end{array}$$

where *S*_*t*_ and *S*_*t*−*m*_ are the skin images of the same candidate location in (7) at time *t* and *t* − *m*, and *b* is a threshold.

For each remaining candidates *X*_*t*_^*n*^ we calculate the motion parameters *p*(*X*_*t*_^*n*^|*X*_*t*−1_) as Gaussian distribution

(9) $$\begin{array}{c} p\left(X_{t}^{n}\;|\;X_{t-1}\right)=N\left(X_{t}^{n};X_{t-1},\Psi \right), \end{array}$$

where Ψ is a diagonal covariance matrix of the standard deviations of location and scale. The likelihood *C*_*t*_^*n*^ of each *X*_*t*_^*n*^ is an accumulation of confidence *C*_*r*_^*s*^ of superpixels *r* located inside *X*_*t*_^*n*^

(10) $$\begin{array}{c} C_{t}^{n}=\frac{S\left(X_{t}^{n}\right)}{S\left(X_{t-1}\right)}\times \underset{r\in \left[1,P\right]}{\sum }C_{r}^{s}, \end{array}$$

where *S*(*X*_*t*_) is the scale of hand *X*_*t*_ and the hand is detected as the best candidate according to the maximum a posteriori (MAP) estimate:

(11) $$\begin{array}{c} X_{t}=\underset{X_{t}^{n}}{\arg}\;\max\;p\left(X_{t}^{n}\;|\;X_{t-1}\right)C_{t}^{n}. \end{array}$$

**Figure 7:**
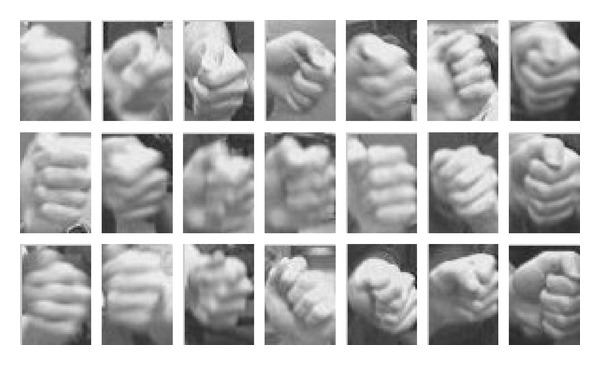
Gesture hand templates.

As we have discussed, the SPT may fail when* occlusion* or* background confusion* occurred. We recover from both failures to give more precise tracked hand and provide the positive samples to ensure updating with correct information. The only case discarded for sampling in our solution is the gesturing hand moves out of the frame, as shown in Figure 5(c). In our failure recovery process, we use the template matching to find the best match from the candidates.  Figure 7 shows some hand templates which are automatically sampled during tracking with the occlusion rate of detection lower than a threshold. Compared to SPT which used only one hand template from the first frame, our template matching is adapted to different hand appearance to recover from the failure.

With remaining *M* sample candidates after discarding and *N* hand templates, we calculate the similarity between each pair of candidate and template using (4). And the best candidate matched to a hand template can be selected with maximum in *M* × *N* similarity matrix.  Figure 3(b) shows an example of* occlusion* recovery which occurred in Figure 3(a); we can see that the hand location is more precisely detected, and the annotation “*Severe Occlusion*” indicated that it is a track result recovered from* occlusion* failure. We consider that the problem of* background confusion* occurs when the standard deviation of the recent *L* detected hand locations below a threshold *T*:

(12) $$\begin{array}{c} \text{std}\left(\overset{L}{\underset{i=t-L+1}{X}}\right)<T. \end{array}$$

Then we trace back to the time *t* − *L* + 1 and retrack each of *K* frames (*K* < *L* and *K* ≤ *H*, where *H* is the number of stored sampling frames used for updating the model) with the same method as for* occlusion* recovery. The appearance model may be updated with all samples from the period of* background confusion* which occurred (e.g., *L*/*U* > *H* and *L* > *W*,  *U* is the frequency of sampling and *W* is the frequency of updating), so we temporally set *U* = 1 and train the new model with all detections from the recovery of* background confusion*. The second row of Figure 4 shows an example of recovery of* background confusion*. Our proposed adaptive superpixel based hand tracking method tracks a frame in about 2.1 seconds with an Intel i7 CPU and 4 GB memory PC running Windows 7, where the SLIC segmentation is the main time consuming process.

The* First-In-First-Out* (FIFO) sampling strategy is used in SPT to discard the outdated hand detections, which may prematurely delete samples with high confidence. We try the deletion of samples considering the confidence of current detection, for chronologically stored samples *S*_1_,…, *S*_*H*_; the sample *S*_*h*_ with confidence *C*_*h*_ is replaced by *X*_*t*_ with confidence *C*_*t*_ if *S*_*h*_ meets

(13) $$\begin{array}{c} \max\left(\frac{1}{2^{h}}\times \frac{H-h+1}{H}\times \frac{1}{C_{h}}\right),\;h=1,\ldots ,H \end{array}$$

which indicates that the early sample (smaller *h*) and sample with smaller confidence has more probability to be replaced. The new hand appearance models is retrained by performing mean shift clustering on updated sample set and recalculated the target-background confidence using (5).

Our adaptive superpixel based hand gesture tracking solution is summarized as in Algorithm 2.

## 4. Gesture Classification

With the gesture motion trajectories tracked by our proposed adaptive superpixel based hand gesture tracking algorithm, the normalized feature vector is extracted from motion trajectory for classifying the hand gesture. We applied multiclass support vector machines (SVM) to classify the gestures due to its property of discrimination on nonlinearly separable feature and efficiency. The duration of the hand gestures depends on their complexity, which caused the tracked motion trajectories with different lengths. We employed the B-form Spline approximation to interpolate the trajectories to a uniformed length as the SVM deals with feature instances of the unified dimension. Given a 2D trajectory with *N* points {*X*_*i*_, *Y*_*i*_}_*i*=1_^*N*^, we interpolate the two dimensions *X*_*i*_ and *Y*_*i*_ to *N*_1_ points independently. For the case of *X*_*i*_, we approximate the function defined by {*i*, *X*_*i*_}_*i*=1_^*N*^ to a piecewise polynomial function *f*(*x*) with order *n*:

(14) $$\begin{array}{c} f\left(x\right)=a_{1}+a_{2}x+\cdots +a_{n}x^{n-1}=\overset{n}{\underset{i=1}{\sum }}a_{i}x^{i-1}. \end{array}$$

A Spline is a smoothed piecewise polynomial function that an interval [*a*, *b*] (e.g., [1, *N*]) is divided into sufficiently small intervals [*ξ*_*i*_, *ξ*_*i*+1_] with *a* = *ξ*_1_ < ⋯<*ξ*_*i*+1_ = *b*. In each interval, a polynomial *f*_*i*_ of low degree can provide a good approximation to corresponding {*i*, *X*_*i*_}_*i*=1_^*N*^. The *B*-form Spline describes the polynomial function as a weighted sum of order *k*:

(15) $$\begin{array}{c} f\left(t\right)=\overset{n}{\underset{i=1}{\sum }}B_{i,k}\left(t\right)\cdot a_{i}. \end{array}$$

Each *B*_*j*,*k*_ is defined on an interval [*ξ*_*i*_, *ξ*_*i*+1_] and is zero elsewhere; *t* is called* knots* and is provided based on the smoothness required. B-splines are functions that

(16) $$\begin{array}{c} \overset{n}{\underset{i=1}{\sum }}B_{j,k}\left(x\right)=1,\;x\in \left[t_{k},t_{n+1}\right]. \end{array}$$

**Figure 8:**
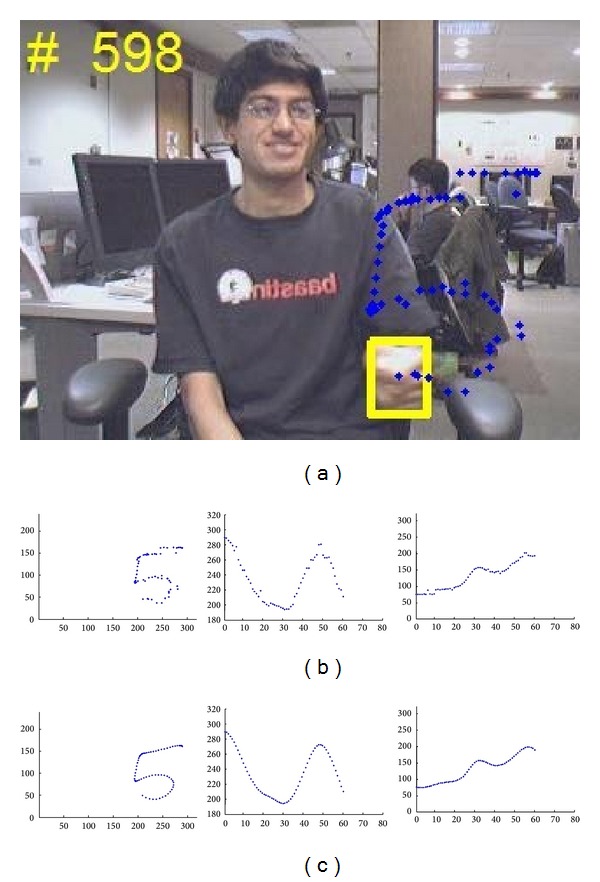
Trajectory interpolation. (a) Accumulated trajectory on the last frame of gesture “5”; (b) tracked trajectory; (c) interpolated and smoothed trajectory. Columns from left to right are trajectory plot with gesture's (*X*, *Y*); plot *X* of gesture trajectory; plot *Y* of gesture trajectory.

Figure 8 shows an example of trajectory interpolation on hand signed digit gesture “5”. The second row shows the original tracked hand positions (60 points) and the third row shows the interpolated and smoothed trajectory (64 points). The first column is combined result of second and third columns, which are the interpolation of *X* and *Y* independently. We further normalize the trajectory points into the range of [0, 1] as

(17) $$\begin{array}{c} X_{i}=\frac{X_{i}-\min{\left\{X\right\}}_{1}^{N_{1}}}{w},\;\;Y_{i}=\frac{Y_{i}-\min{\left\{Y\right\}}_{1}^{N_{1}}}{h}, \end{array}$$

where *w* and *h* are the sizes of the video frame.

We employed the SVM library from [16] for our multiclass hand gesture trajectories classification task, which used one-against-one approach to construct *k*(*k* − 1)/2 classifiers that *k* is the number of gesture classes. A simple voting strategy is applied to decide the class of an input sequence in test. The two parameters *c* (cost of the quadratic problem) and *g* (gamma of RBF kernel) are optimized with 3-fold cross validation in the training set.

## 5. Experimental Results

**Figure 9:**
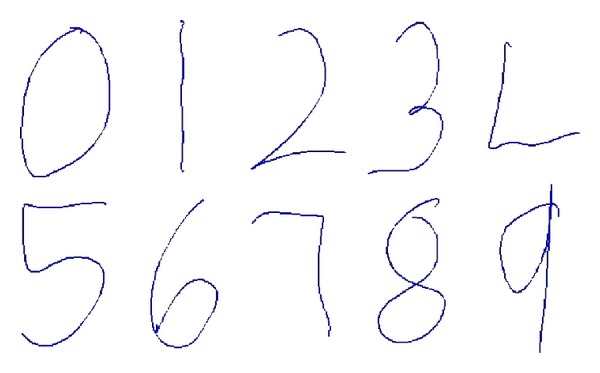
Ten classes of hand signed digit gestures.

**Figure 10:**
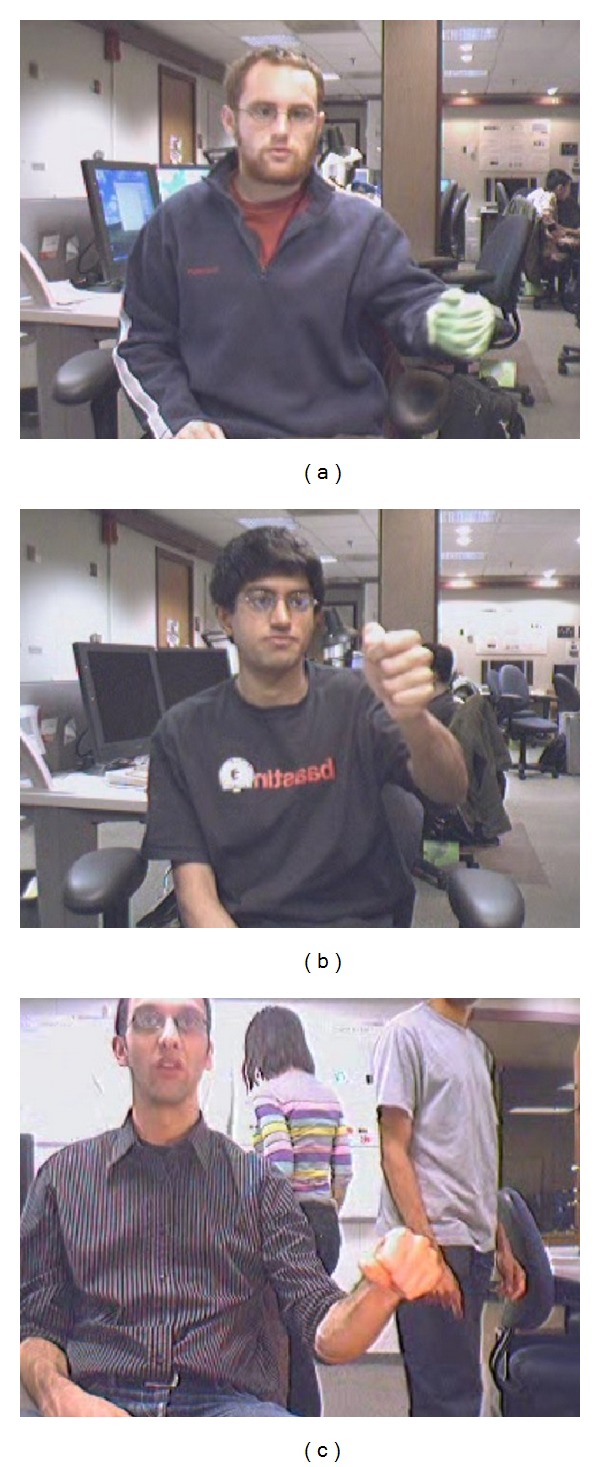
Sample frames from three gesture set.

In this section, our proposed adaptive superpixel based hand gesture tracking and recognition system were evaluated on the hand signed digit gesture dataset provided by Alon et al.'s work [1]; the dataset defined 10 classes of gesture from digit 0 to digit 9; Figure 9 gives a trajectory example for each class which is tracked with our Adaptive Superpixel Hand Tracking algorithm. There are three sets contained in the dataset, the* training set*, the* easy set,* and the* hard set*. We use only the* easy set* and the* hard set*, as the users in the* training set* (e.g., example frame in Figure 10(a)) wore colored gloves and long sleeve which simplifies the tracking from the confusion of skin-like objects. We do the cross validation inside the* easy set *(Figure 10(b)) and* hard set* (Figure 10(c)) to measure the performance of the system.

### 5.1. Easy Test Set

The easy test set contains 30 video sequences, three from each of 10 users which are captured in office environment. The user signed each of 10 gestures once and wore short sleeves; totally there are 300 gesture instances in this set.

Firstly we use one sequences from each user for SVM training (100 gestures that 10 for each class), and test on the remaining sequences (200 gestures that 20 for each class). By switching the training/test video sequences, there are three tests.  Table 1 gives the confusion matrix of the recognition results. The number of correctly and falsely recognized gestures for each class is accumulated from the three tests. The first row is the ground truth labels of gesture classes, and the first column is the recognized class labels. We see that totally 5 gestures are falsely classified out of 600 gestures from three tests; the recognition accuracy is 595/600 = 99.17%.

Similarly, we use two sequences from each user for SVM training (200 gestures that 20 for each class) and test on the remaining sequences (100 gestures that 10 for each class). There are totally 4 gestures misclassified out of 300 gestures from three tests. The recognition rate is 296/300 = 98.67%.  Table 2 gives the confusion matrix of the results.

### 5.2. Hard Test Set

The hard test set contains 14 sequences, two from each of seven users; totally there are 140 gesture instances in this set. In this set there are one to three distractors moving around the gesturing user (see Figure 10(c)). We use half of the data (one sequence from each user, 70 gestures with 7 from each class) to train the SVM and test on the remaining. There are two tests by switching the training/test data.  Table 3 shows the confusion matrix of recognition result for each class; there are only 2 gestures misclassified out of 140 gestures; the recognition accuracy is 138/140 = 98.57%.

We also compared our approach with the state of the art methods as shown in Table 4. To the best of our knowledge, we have referenced all publications that experiment the gesture recognition on the Alon et al.'s dataset [1]. We state that our hand gesture recognition approach outperforms the other solutions with significant improvement, which benefit mainly from our reliable hand motion tracking solution in long sequences.

## 6. Conclusion

We proposed an adaptive superpixel based hand gesture tracking and recognition system in this paper to address the gestures expressed by human hand motion trajectories. With the target hand detected in first few frames using SLIC segmentation and motion subtraction and then refined by segmented object regions of CTM, our adaptive hand motion tracking well handles the occlusion and background confusion problem. The trajectory classification using SVM models on hand signed digit gestures gives promising results. Experimental results show that our proposed system can achieve better performance compared to the existing state of the art methods with the recognition accuracy 99.17% for easy set and 98.57 for hard set. Future works may focus on multiobjects or two-hand gesture tracking system.

## Figures and Tables

**Algorithm 1:**
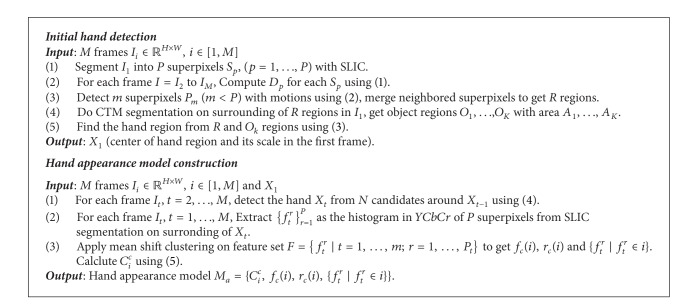
Initial hand detection and model construction.

**Algorithm 2:**
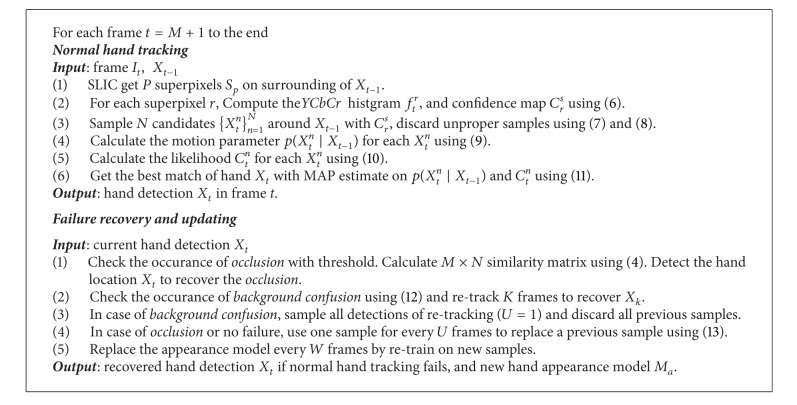
Adaptive superpixel based hand gesture tracking.

**Table 1:** Confusion matrix of recognition result on easy set, using 1/3 data for training and 2/3 for testing. Gestures counts are accumulated from three tests by switch training/test data.

	0	1	2	3	4	5	6	7	8	9
0	*60 *	0	0	0	0	0	2	0	0	0
1	0	*60 *	0	0	0	0	0	0	0	0
2	0	0	*60 *	0	0	0	0	0	0	0
3	0	0	0	*60 *	0	0	0	0	0	0
4	0	0	0	0	*59 *	0	0	0	0	0
5	0	0	0	0	0	*60 *	0	0	0	1
6	0	0	0	0	0	0	*58 *	0	0	0
7	0	0	0	0	0	0	0	*59 *	0	0
8	0	0	0	0	0	0	0	0	*60 *	0
9	0	0	0	0	1	0	0	1	0	*59 *

False	0	0	0	0	1	0	2	1	0	1

**Table 2:** Confusion matrix of recognition result on easy set, using 2/3 data for training and 1/3 for testing. Gestures counts are accumulated from three tests by switch training/test data.

	0	1	2	3	4	5	6	7	8	9
0	*30 *	0	0	0	0	0	1	0	0	0
1	0	*30 *	0	0	0	0	0	0	0	0
2	0	0	*30 *	0	0	0	0	0	0	0
3	0	0	0	*30 *	0	0	0	1	0	0
4	0	0	0	0	*29 *	0	0	0	0	0
5	0	0	0	0	0	*30 *	0	0	0	1
6	0	0	0	0	0	0	*29 *	0	0	0
7	0	0	0	0	0	0	0	*29 *	0	0
8	0	0	0	0	0	0	0	0	*30 *	0
9	0	0	0	0	1	0	0	0	0	*29 *

False	0	0	0	0	1	0	1	1	0	1

**Table 3:** Confusion matrix of recognition result on hard set, using 1/2 data for training and 1/2 for testing. Gestures counts are accumulated from two tests.

	0	1	2	3	4	5	6	7	8	9
0	*14 *	0	0	0	0	0	2	0	0	0
1	0	*14 *	0	0	0	0	0	0	0	0
2	0	0	*14 *	0	0	0	0	0	0	0
3	0	0	0	*14 *	0	0	0	0	0	0
4	0	0	0	0	*14 *	0	0	0	0	0
5	0	0	0	0	0	*14 *	0	0	0	0
6	0	0	0	0	0	0	*12 *	0	0	0
7	0	0	0	0	0	0	0	*14 *	0	0
8	0	0	0	0	0	0	0	0	*14 *	0
9	0	0	0	0	0	0	0	0	0	*14 *

False	0	0	0	0	0	0	2	0	0	0

**Table 4:** Comparsion with state of arts on the same datasets.

Approach	Accuracy of easy set (%)	Accuracy of hard set (%)
Correa et al. [17]	75.00	N/A
Malgireddy et al. [18]	93.33	N/A
Kulkarni [19]	N/A	80.71
Yao and Li [20]	95.67	86.43
Hanson [21]	100	76.40
Alon et al. [1]	94.60	85.00
**Our**	**99.17**	**98.57**
